# WHAT ABOUT HISPANIC SERVING INSTITUTIONS: THE ROLE OF MICRO-CREDENTIALS AMONG MINORITY-SERVING INSTITUTIONS

**DOI:** 10.1093/geroni/igae098.1004

**Published:** 2024-12-31

**Authors:** Patricia Heyn

**Affiliations:** Marymount University, Fairfax Station, Virginia, United States

## Abstract

Hispanic-Serving Institutions (HIS) are educational institutions that have an enrollment of at least 25% of undergraduate students and constitute one of the fastest-growing categories in higher education. Here, we explore the benefits and implications of micro-credential certifications at Hispanic-Serving Institutions, with the goal of uncovering their potential as transformative tools in addressing educational and workforce disparities faced by Hispanic learners and trainees. We cannot underscore the strengths of micro-credential programs, such as enhancing access to post-secondary education for Hispanic learners, offering flexible educational pathways that are respectful and sensitive to their cultural and life commitments, and directly linking academic achievements with marketable skills in the workforce. Importantly, the alignment of micro-credentials with high-demand career fields offers a promising avenue for improving the economic outcomes of Hispanic graduates. Yet, there are still critical challenges to consider, including the need for broader recognition of micro-credentials in the professional market, concerns about the digital divide affecting equitable access to these programs, and the institutional capacities of HSIs to support high-quality micro-credential offerings. Comprehensive research, evaluation systems, and evidence-based guidance are needed to optimize micro-credentials at HSIs, suggesting research funding and policy adjustments, infrastructure investments, and collaborative initiatives with both academic centers and industry partners to ensure these programs effectively contribute to closing the educational, professional, and economic gaps faced by Hispanic communities.

